# Corrosion Behavior of Ti–Nb–Zr–Si Alloy after One-Year Exposure to Simulated Inflammatory Conditions

**DOI:** 10.1021/acsomega.6c07507

**Published:** 2026-09-24

**Authors:** Aydin Bordbar-Khiabani, Michael Gasik

**Affiliations:** † 3259 VTT Technical Research Centre of Finland Ltd., Tekniikantie 21, Espoo 02044, Finland; ‡ Department of Chemical and Metallurgical Engineering, School of Chemical Engineering, Aalto University, Espoo 02150, Finland

## Abstract

Long-term corrosion and elemental release are critical factors controlling the clinical reliability of titanium-based implants, particularly under inflammatory conditions. In this study, commercially pure titanium (CP-Ti), Ti–6Al–4V, and Ti–Nb–Zr–Si (TNZS) alloy were immersed for 372 days under static, non-refreshed conditions in media initially prepared to represent normal, inflammatory, and severe inflammatory environments. After exposure, the specimens were removed, rinsed, and evaluated by open-circuit potential, potentiodynamic polarization, and electrochemical impedance spectroscopy in freshly prepared solutions of the corresponding nominal compositions. The original immersion solutions were analyzed by inductively coupled plasma optical emission spectrometry to determine released elements. TNZS maintained the most noble electrochemical behavior, lowest corrosion current density, and highest passive-layer resistance in all media. Elemental release increased with solution aggressiveness, following the order normal < inflammatory < severe inflammatory. Ti–6Al–4V released Al and V, whereas TNZS released lower total ion concentrations and avoided potentially harmful alloying elements. Under the tested static, non-refreshed conditions, TNZS showed lower apparent corrosion current density, higher fitted passive-layer resistance, and lower filtered dissolved elemental release than CP-Ti and Ti–6Al–4V.

## Graphical abstract

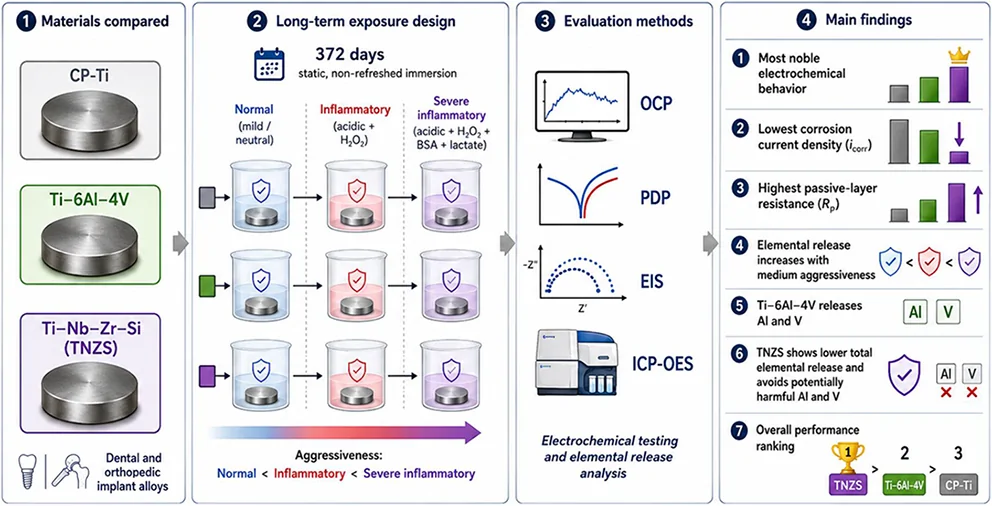

## 1 Introduction

Titanium and its alloys remain central materials for dental and orthopedic implants because they combine high specific strength, good fatigue resistance, and spontaneous passivation in physiological fluids.^1,2^ Their apparent bioinertness, however, should not be interpreted as complete electrochemical inactivity.^3,4^ Once implanted, metallic biomaterials are exposed to a dynamic biological environment in which proteins, inorganic ions, inflammatory cells, reactive oxygen species, and local pH variations continuously interact with the surface.^5^ Under such conditions, the passive oxide film may undergo repeated cycles of dissolution, repassivation, hydration, and chemical modification.^6,7^ These processes are especially important during long-term implantation, where even low corrosion rates can lead to measurable accumulation of released ions and corrosion products at the tissue–implant interface.

Conventional CP-Ti and Ti–6Al–4V have been widely used in clinical applications, but each material has limitations under aggressive biological conditions.^8^ Both rely mainly on a TiO_2_-based passive film, which may be altered by inflammatory oxidants such as H_2_O_2_ and acidic environments.^9–11^ Ti–6Al–4V provides higher mechanical strength, but the possible release of Al and V remains a concern for long-term applications, particularly when local inflammation, infection, or mechanically assisted corrosion accelerates degradation.^12,13^ These concerns have encouraged the development of Al- and V-free β-type titanium alloys containing more biocompatible alloying elements such as Nb and Zr. In this context, Ti–Nb–Zr-based alloys are attractive because Nb and Zr can improve passive-film stability while avoiding the biological concerns associated with Al and V.^14^

A Ti–Nb–Zr–Si alloy, referred to here as TNZS, was recently developed as a candidate metallic biomaterial and compared with CP-Ti and Ti–6Al–4V under normal, inflammatory, and severe inflammatory conditions.^15^ That study showed that TNZS formed a microstructure containing α-Ti, β-Ti, and silicide intermetallic phases, and that these phases contributed to improved electrochemical behavior in acidic H_2_O_2_-containing environments. The same work also reported better biological response of TNZS compared with Ti–6Al–4V, including improved HOC and HUVEC behavior and stronger osteogenic marker expression.^15^

Although short-term electrochemical and biological responses provide valuable information, they do not fully represent the long-term behavior of implant alloys after extended contact with physiological or inflammatory fluids.^16^ *In vivo*, implants may remain in contact with stagnant or slowly refreshed biological fluids for prolonged periods, especially in confined peri-implant regions.^17^ During this time, solution chemistry can change, reactive species may decompose, proteins may adsorb or denature, and released ions may accumulate.^18^ Therefore, evaluating the electrochemical response only after short immersion times may underestimate or misrepresent the condition of the passive layer after long-term exposure.

Long-term ion release is also clinically relevant because corrosion products are not distributed uniformly throughout the body immediately after release.^19^ Instead, they are first generated locally near the implant surface, where they may interact with macrophages, osteoblasts, endothelial cells, proteins, and extracellular matrix components.^20^ Even when the total released mass is low compared with systemic exposure limits, local accumulation may influence inflammatory signaling, osteogenesis, angiogenesis, and peri-implant tissue stability.^21^ Therefore, corrosion resistance and biological performance should be interpreted together with elemental release data.

A major research gap remains because most electrochemical studies on titanium biomaterials are limited to short immersion periods, typically from a few hours to several days or weeks. Previous studies have provided important insights into time-dependent passivation of Ti and Ti–6Al–4V in Hank’s solution up to 168 h,^22^ Ti–6Al–7Nb and Ti–6Al–4V ELI in SBF up to 360 h,^23^ β-type Ti–Nb–Ta alloys over 15 days,^24^ and Ti–6Al–4V in H_2_O_2_/albumin-containing media up to 120 h.^25^ More recent long-term studies on Ti–6Al–4V have extended exposure to three months for surface-modified alloys and to longer periods for additively manufactured scaffolds or retrieved orthodontic mini-implants.^26–28^ However, direct one-year static immersion data comparing CP-Ti, Ti–6Al–4V, and a newly developed TNZS alloy under normal, inflammatory, and severe inflammatory media are still lacking.

This study investigates whether TNZS alloy can maintain stable corrosion resistance and low elemental release after approximately one year of exposure to aggressive simulated biological environments. By integrating long-term immersion, post-exposure electrochemical testing, and ion-release analysis, this work provides a more clinically relevant assessment of TNZS electrochemical stability compared with conventional titanium biomaterials. It also clarifies whether the short-term corrosion and biological advantages of TNZS persist after prolonged exposure.

## 2 Materials and Methods

### 2.1 Sample Preparation

The same CP-Ti, Ti–6Al–4V, and TNZS materials used in the previous study were used in the present long-term immersion investigation.^15^ The chemical composition of the TNZS alloy was Ti balance, 11.15 ± 0.19 wt % Zr, 9.61 ± 0.32 wt % Nb, and 1.23 ± 0.20 wt % Si. Commercially pure titanium contained 99.6% Ti, while Ti–6Al–4V contained 5.5–6.76 wt % Al, 3.5–4.5 wt % V, and Ti as the balance. The earlier microstructural and phase characterization showed that the TNZS alloy consisted of α-Ti, β-Ti, and intermetallic silicide phases, with lamellar eutectic regions distributed within the metallic matrix (Figure S1).^15^ The specimens were prepared as discs with a diameter of 15 mm and a thickness of 0.5 mm. Before immersion, the surfaces were ground using 240–2500 grit SiC papers, polished with 0.3 μm alumina suspension, ultrasonically cleaned in 70% ethanol and deionized water, and dried before use.

### 2.2 Electrochemical Measurements

All specimens were immersed in three simulated physiological media: normal, inflammatory PBS/H_2_O_2_/HCl solution adjusted to pH 5, and severe inflammatory PBS/H_2_O_2_/BSA/lactate/HCl solution adjusted to pH 3. The composition of each medium is summarized in Supplementary Table ST1. Specimens were placed in plastic bottles containing 15 mL of the corresponding solution. The bottles were closed and maintained under static, non-refreshed conditions in a dark box at ambient laboratory temperature for 372 d. The solutions were neither refreshed nor supplemented during the 372 d exposure. Residual H_2_O_2_ concentration, dissolved oxygen, ORP, BSA stability, and lactate concentration were not monitored during immersion. Consequently, the terms “inflammatory” and “severe inflammatory” refer to the initial formulations of the media and do not imply that their original chemical compositions remained constant throughout the exposure period.

This protocol was designed to evaluate long-term surface aging and ion release under closed, static, non-refreshed exposure conditions, including the effects of solution aging and accumulation of released species. Moreover, the present work was intentionally designed as a long-term static baseline study rather than a complete simulation of the dynamic peri-implant environment. This approach enables comparison of the intrinsic chemical aging response of the three materials under identical exposure conditions, while dynamic effects such as fluid renewal, reactant replenishment, removal of corrosion products, and mass transport are excluded. These factors should be incorporated in subsequent long-term studies to evaluate the behavior of the materials under more physiologically representative conditions.

After 372 d of immersion at ambient temperature, the specimens were removed from the aged solutions, gently rinsed with deionized water to remove loosely attached residues, and immediately used for electrochemical measurements in freshly prepared solutions with the same nominal composition as the corresponding immersion medium. The freshly prepared electrochemical test solutions were maintained at 37 ± 0.5 °C throughout the electrochemical measurements. Electrochemical measurements were performed using an IviumStat.h potentiostat (Ivium Technologies, The Netherlands) in a conventional three-electrode cell. A graphite rod was used as the counter electrode, Ag/AgCl as the reference electrode, and the Ti-based specimen as the working electrode, with an exposed area of 1 cm^2^. All electrochemical measurements were performed in triplicate.

OCP was recorded for a fixed period of 600 s to monitor the initial short-term potential response of the 372 d aged surfaces after transfer to the freshly prepared corresponding electrolytes. This interval was applied consistently to all specimens and was selected to maintain methodological comparability with our previous study,^15^ in which the same materials and nominal solution formulations were evaluated after 1 and 12 h of exposure.

PDP tests were performed from −1.5 to +1.5 V versus OCP at a scan rate of 1 mV·s^–1^. Since the anodic branches of the titanium-based specimens were strongly influenced by passive-film formation and did not exhibit a well-defined linear Tafel region, the corrosion current density was estimated by extrapolating the cathodic Tafel region to the corrosion potential. Therefore, only the |β_c_| is reported.

EIS was carried out at OCP using a 10 mV AC amplitude over a frequency range of 100 kHz to 0.010 Hz. EIS data were analyzed using ZView software (version 3.1, Scribner Associates), and the reliability of the fitting was verified by chi-squared (χ^2^) values on the order of 10^–3^.

### 2.3 Ion Release Assessment

After removal of the specimens, the remaining one-year immersion solutions were collected for ion-release analysis. The solutions were filtered before analysis to remove suspended particles, corrosion debris, or detached surface products. The concentrations of released elements were measured by inductively coupled plasma optical emission spectrometry (ICP-OES) using a PerkinElmer DV5300 instrument. The filter membranes and retained residues were not digested or analyzed. Therefore, the reported concentrations represent the ICP-OES-detectable elemental fraction present in the filtered solutions and should not be interpreted as total material release or total mass loss from the specimens. The analyzed elements were selected according to the composition of each material: Ti for CP-Ti; Ti, Al, and V for Ti–6Al–4V; and Ti, Nb, Zr, and Si for TNZS. The elemental concentrations were reported in μg·L^–1^.

The pH of each immersion solution was also measured after the 372 d exposure period to determine the change in solution chemistry during long-term immersion. For each material and medium, the ICP-OES and pH results were reported as mean ± standard deviation from three measurements. The immersion protocol was designed as an adapted long-term static ion-release assay rather than a short-term standard extraction test. The use of 15 mL solution ensured complete immersion of the specimens and provided sufficient volume for filtration, pH measurement, and ICP-OES analysis. The measured elemental concentrations represent cumulative end point concentrations in the filtered 15 mL immersion solutions. They reflect the combined effects of release, accumulation, possible adsorption, precipitation, complexation, and retention of material on the specimen or filter. Because intermediate sampling was not performed, the data cannot be used to establish linear release kinetics or a constant release rate over 372 d.

All immersions were performed in metal-free polypropylene tubes instead of glass containers to minimize possible background contamination. This precaution was necessary because borosilicate or soda-lime glass may leach Si, Na, B, Al, and other trace elements during prolonged immersion, particularly under acidic conditions and in the presence of H_2_O_2_.

## 3 Results and Discussion

### 3.1 Electrochemical Characterization

#### 3.1.1 Open Circuit Potential

Figure 1 shows OCP evolution of specimens after 372 d of immersion in different simulated physiological conditions. In the normal PBS solution, all specimens showed relatively stable OCP curves during the 600 s measurement. The final potentials after one year were approximately −0.096 V for CP-Ti, −0.036 V for Ti–6Al–4V, and −0.012 V for TNZS versus Ag/AgCl. Compared with the 12 h results of the previous study, these values shifted slightly in the noble direction, suggesting some stabilization or reconstruction of the passive film during long-term exposure to PBS. The positive shift was more pronounced for CP-Ti and Ti–6Al–4V, while TNZS showed only a small change, probably because its passive film had already reached a relatively stable condition after short-term immersion.

**Figure 1:**
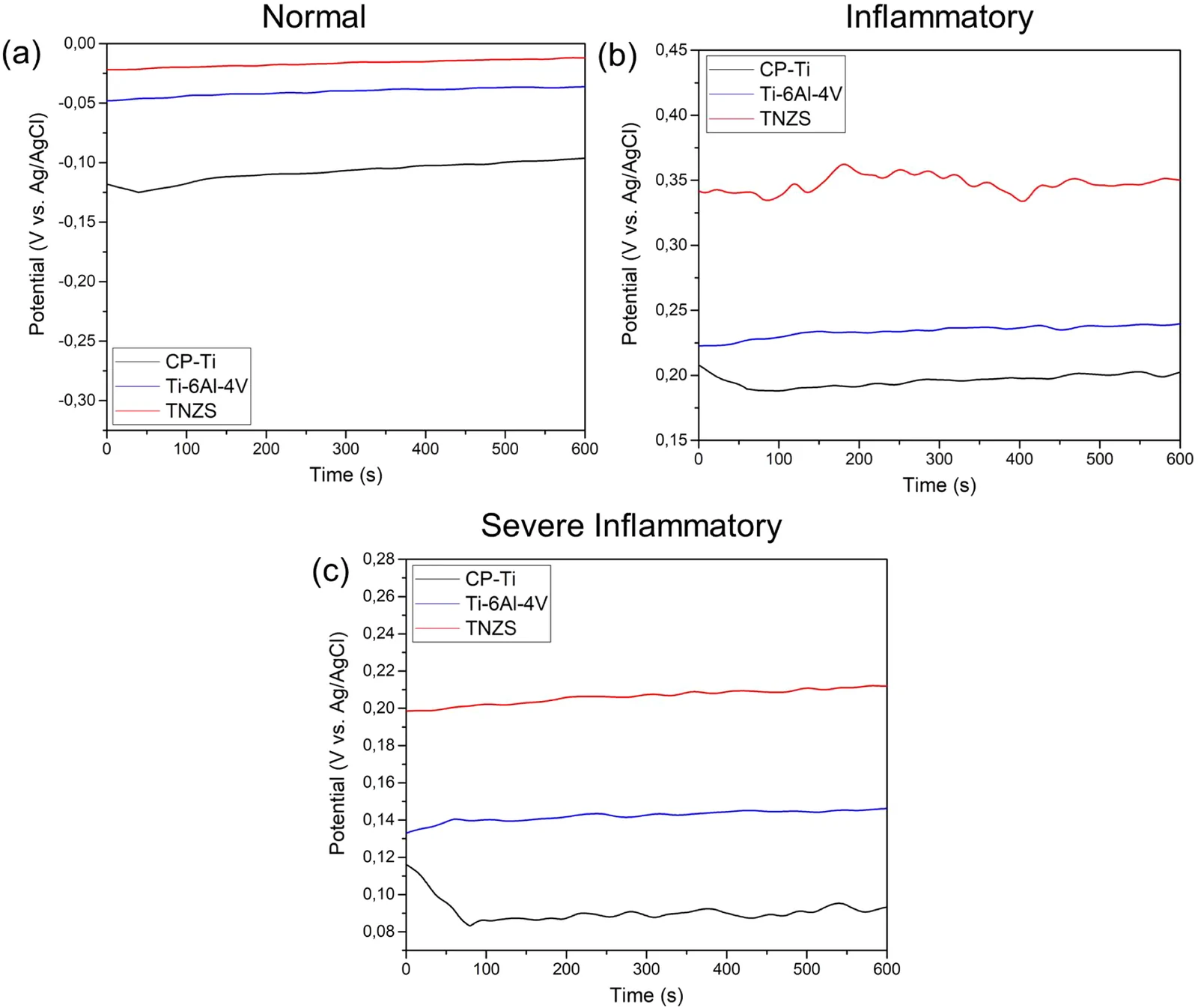
OCP evolution of CP-Ti, Ti–6Al–4V, and TNZS after 372 d of immersion in different simulated physiological conditions: (a) normal PBS, (b) inflammatory PBS/H_2_O_2_/HCl solution, and (c) severe inflammatory PBS/H_2_O_2_/BSA/lactate/HCl solution.

The inflammatory medium produced a clear positive shift in OCP compared with PBS. After one year, the final potentials were approximately 0.202 V for CP-Ti, 0.240 V for Ti–6Al–4V, and 0.350 V for TNZS. This electropositive shift confirms the oxidizing effect of H_2_O_2_-containing PBS. H_2_O_2_ can act as an additional cathodic reactant and may generate oxidizing intermediates that adsorb on the titanium passive film, leading to higher OCP values. However, the one-year inflammatory potentials of Ti–6Al–4V and TNZS were slightly lower than their 12 h values. The slightly lower OCP values observed after one-year exposure compared with the short-term condition should not be interpreted as direct evidence of a measured residual H_2_O_2_ concentration. Because residual H_2_O_2_, dissolved oxygen, and ORP were not monitored in the aged immersion media, H_2_O_2_ decomposition or consumption can only be considered a plausible contributing factor. Other factors, including passive-film hydration, surface contamination, adsorption of solution species, released-ion accumulation, and changes in interfacial chemistry during long-term static exposure, may also have contributed. Therefore, the OCP results are interpreted as the response of aged surfaces during re-exposure to freshly prepared media of the same nominal composition, not as the in-situ mixed potential inside the aged one-year bottles.

The behavior of TNZS in the inflammatory solution was particularly important. Although its final one-year OCP was slightly lower than the short-term 12 h value, it remained clearly higher than those of CP-Ti and Ti–6Al–4V. This indicates that the passive film on TNZS retained better stability under oxidative acidic conditions. The more positive end-of-monitoring potential of TNZS is consistent with a different aged interfacial state from those of CP-Ti and Ti–6Al–4V. A possible contribution from Nb-, Zr-, and Si-containing surface oxides is supported by previous studies;^15^ however, the composition, oxidation states, and structure of the aged TNZS surface were not measured here. Therefore, the origin of the OCP difference cannot be established from the present data alone.

In the severe inflammatory solution, the OCP values were lower than those in the inflammatory medium but higher than those in PBS. The final potentials were approximately 0.093 V for CP-Ti, 0.146 V for Ti–6Al–4V, and 0.212 V for TNZS. Thus, the environmental order was normal < severe inflammatory < inflammatory. This trend agrees with the previous short-term study, where the addition of BSA and lactate to the H_2_O_2_-containing solution reduced the OCP. BSA and lactate-containing species can adsorb on the titanium surface, block cathodic sites, and decrease the rate of H_2_O_2_ reduction, resulting in lower potentials than in the inflammatory medium alone.

Compared with the 12 h condition, the severe inflammatory potentials decreased after one year, suggesting progressive modification of the passive films by low pH, H_2_O_2_, protein, and lactate. This lower OCP should not be interpreted as improved corrosion resistance. Instead, it reflects the combined effects of oxidizing-species depletion, biomolecule adsorption, cathodic-site blocking, and chemical degradation of the passive layer. CP-Ti showed a slight negative drift during the 600 s measurement, indicating continued surface instability, whereas Ti–6Al–4V and TNZS showed more stable behavior. Overall, the OCP results show that TNZS maintained the noblest potential in all media, but the corrosion resistance after one year must be interpreted together with PDP and EIS results, since OCP alone does not directly quantify corrosion rate.

#### 3.1.2 Electrochemical Impedance Spectroscopy

Figures 2 and 3 show the Nyquist, Bode modulus, and Bode phase plots of CP-Ti, Ti–6Al–4V, and TNZS after 372 d of immersion in the normal, inflammatory, and severe inflammatory media. The impedance spectra were fitted using the same equivalent circuit used in the previous short-term study (Figure S2), consisting of the solution resistance, R_s_, in series with a parallel combination of passive-layer resistance, R_pl_, and a constant phase element, CPE. This circuit was previously selected because the EIS response of the tested titanium-based materials showed one dominant time constant, while surface heterogeneity, roughness, oxide defects, and non-ideal capacitive behavior required the use of a CPE instead of an ideal capacitor.

**Figure 2:**
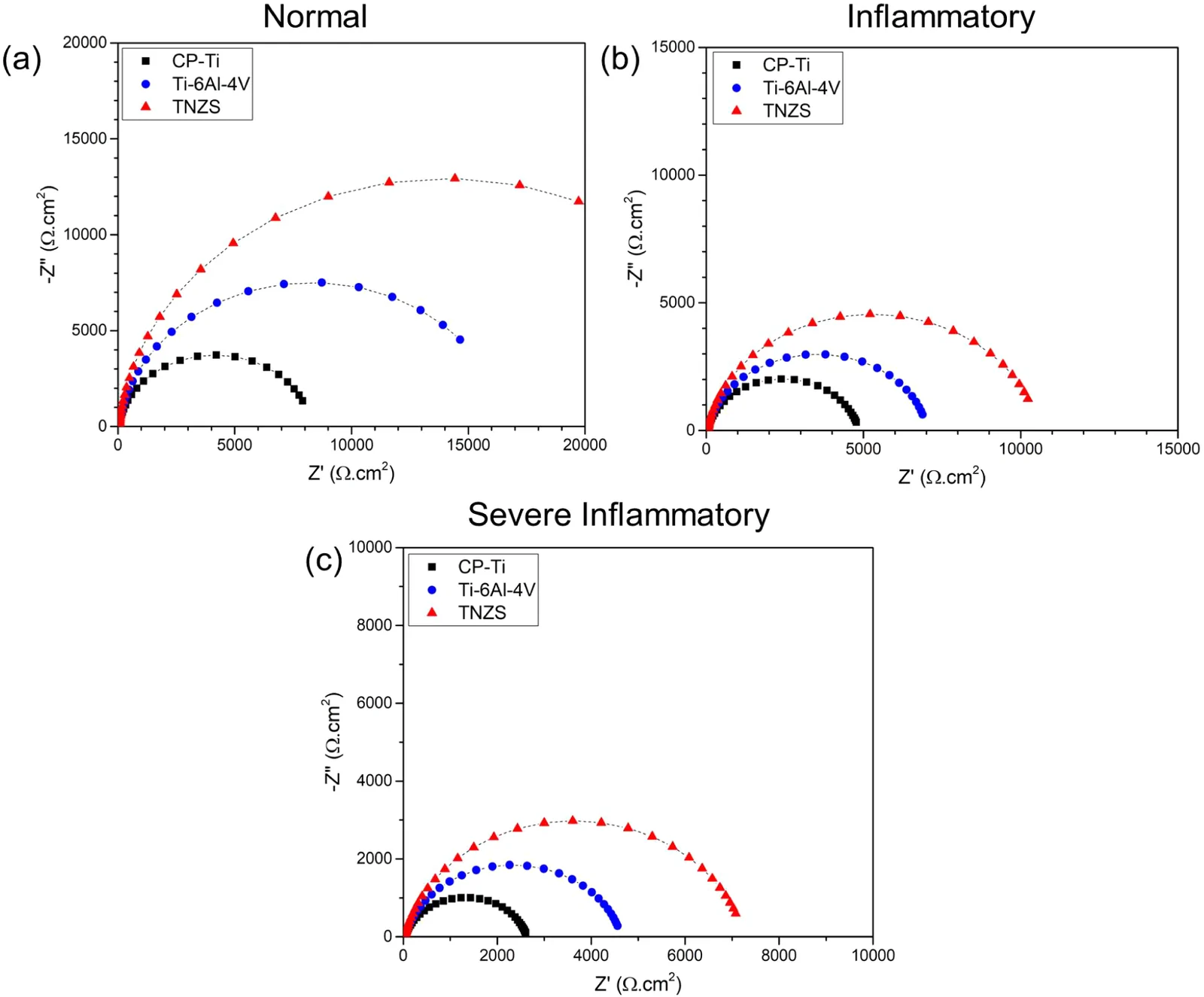
Nyquist plots of CP-Ti, Ti–6Al–4V, and TNZS after 372 d of immersion in different simulated physiological conditions: (a) normal PBS, (b) inflammatory PBS/H_2_O_2_/HCl solution, and (c) severe inflammatory PBS/H_2_O_2_/BSA/lactate/HCl solution.

**Figure 3:**
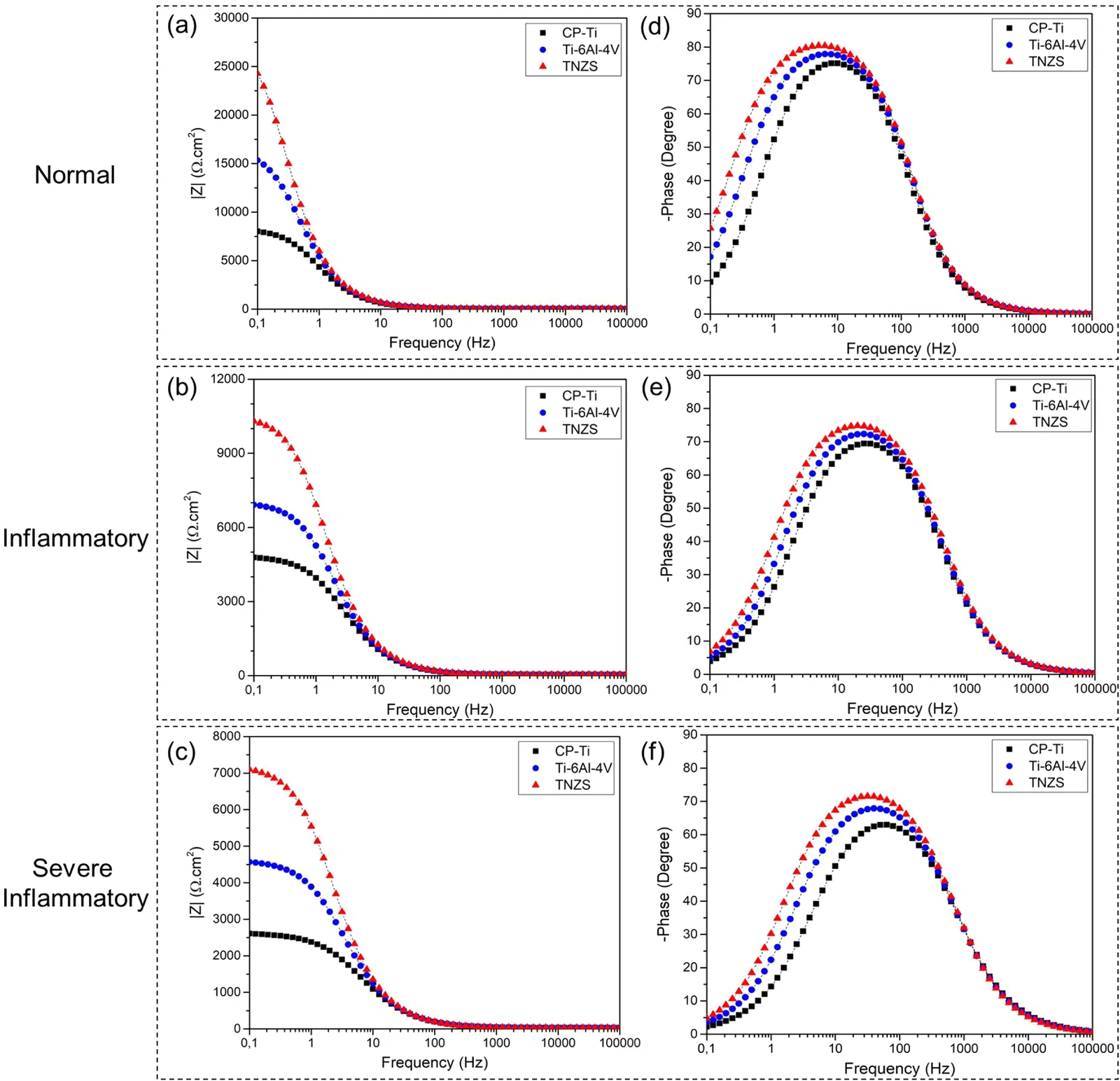
Bode modulus and Bode phase plots of CP-Ti, Ti–6Al–4V, and TNZS after 372 d of immersion in different simulated physiological conditions: (a, d) normal PBS, (b, e) inflammatory PBS/H_2_O_2_/HCl solution, and (c, f) severe inflammatory PBS/H_2_O_2_/BSA/lactate/HCl solution.

The same equivalent circuit used in the previous short-term study was applied to fit the one-year EIS spectra because the impedance response still exhibited one dominant time constant in the Bode phase plots. Although long-term immersion may modify the passive layer through hydration, aging, surface roughening, or adsorption of solution species, these effects were reflected by changes in R_pl_, CPE, and n rather than by the appearance of an additional electrochemical time constant. Therefore, the circuit remained appropriate for comparing the passive-layer behavior after 1 h, 12 h, and 372 d of immersion.

The fitted EIS parameters after one year are summarized in Supplementary Table ST2. In normal, the R_pl_ values were 8.2, 16.5, and 28.0 kΩ·cm^2^ for CP-Ti, Ti–6Al–4V, and TNZS, respectively. The corresponding Nyquist plots showed the largest capacitive loop for TNZS and the smallest one for CP-Ti, indicating that the protective ability of the passive film followed the order TNZS > Ti–6Al–4V > CP-Ti. The Bode modulus plots showed the same trend, with TNZS exhibiting the highest low-frequency impedance. This confirms that, even after long-term immersion, the TNZS alloy retained the most protective surface condition among the three materials.

Compared with the short-term immersion behavior, the one-year PBS data suggest that the passive films did not continue to grow into a highly resistive state indefinitely. Although the short-term study showed an increase in R_pl_ from 1 to 12 h, which was attributed to passive-film formation and reconstruction, the present one-year response remained within the same practical impedance range as the earlier spectra rather than increasing by orders of magnitude. This behavior is reasonable because long-term exposure under static conditions can lead not only to oxide maturation but also to hydration, aging, surface contamination, local defect formation, and partial transformation of the passive film. Therefore, the one-year PBS response should be interpreted as a stabilized long-term passive state rather than simply a continuation of the short-term increase in passive-layer resistance.

In the inflammatory medium, the R_pl_ values decreased to 4.8 kΩ·cm^2^ for CP-Ti, 7.0 kΩ·cm^2^ for Ti–6Al–4V, and 10.5 kΩ·cm^2^ for TNZS. Thus, the passive-layer resistance in the inflammatory medium was approximately 41–42% of the corresponding PBS value for CP-Ti and Ti–6Al–4V, and about 38% for TNZS. This decrease confirms the damaging effect of the acidic H_2_O_2_-containing solution on the passive layers. Short-term studies have associated lower impedance in freshly prepared inflammatory media with reactions between titanium oxide and H_2_O_2_.^15^ In the present long-term static system, similar reactions may have occurred initially, but their contribution over the full exposure period cannot be quantified because the solution chemistry evolved without monitoring.

The inflammatory solution also affected the CPE parameters. The n values decreased from about 0.94–0.95 in PBS to 0.89–0.91 in the inflammatory medium, indicating a greater deviation from ideal capacitive behavior. This suggests that the passive films became more heterogeneous after one year in acidic H_2_O_2_-containing PBS. Such heterogeneity may result from repeated local oxide dissolution and repassivation, peroxide-assisted oxidation, chloride interaction, and formation of a less uniform hydrated oxide layer. Nevertheless, TNZS maintained the highest R_pl_ in this medium, showing that the Nb-, Zr-, and Si-containing passive layer remained more resistant to peroxide-induced degradation than the oxide films on CP-Ti and Ti–6Al–4V.

The severe inflammatory medium produced the lowest impedance response for all materials. The fitted R_pl_ values were 2.6, 4.6, and 7.2 kΩ·cm^2^ for CP-Ti, Ti–6Al–4V, and TNZS, respectively. Compared with PBS, these values represent decreases of about 68% for CP-Ti, 72% for Ti–6Al–4V, and 74% for TNZS. The specimens aged in the medium initially formulated as severe inflammatory showed the lowest post-exposure impedance response. This indicates that the overall one-year exposure history in this solution produced the least protective aged surfaces, although the relative contributions of residual H_2_O_2_, low pH, BSA, lactate, and solution-aging products cannot be separated.

The stronger degradation in the severe inflammatory medium can be explained by several simultaneous effects. The low pH increases the solubility and chemical instability of titanium oxide. H_2_O_2_ promotes oxidative attack and formation of reactive peroxide-related surface species. Lactate can participate in complexation reactions with titanium species, facilitating dissolution or destabilization of the passive film. BSA adsorption can further modify the interface, producing a more heterogeneous surface layer and affecting charge-transfer and capacitive behavior. These combined processes reduce R_pl_ and produce lower phase angles, indicating a less ideal and less protective passive film.

The C_eff_ values calculated from the CPE parameters were in the range of approximately 1.38–3.04 × 10^–5^ F·cm^–2^, while the calculated d_eff_ values ranged from about 1.31 to 2.89 nm. These values are consistent with the previous study, where the calculated effective passive-layer thickness did not exceed a few nanometers. It is important to emphasize that d_eff_ represents the effective thickness of the compact electrochemically active barrier layer, not the total physical thickness of all surface oxides, hydrated products, adsorbed proteins, or deposits accumulated during one year. Therefore, the fact that d_eff_ increased only by a few nanometers is reasonable.

Interestingly, the calculated d_eff_ values were slightly higher in the inflammatory and severe inflammatory media than in PBS, even though R_pl_ decreased. This apparent contradiction is meaningful. A larger calculated d_eff_ does not necessarily indicate a more protective passive film. In aggressive media, lower C_eff_ and higher apparent d_eff_ may reflect the formation of a more hydrated, porous, chemically modified, or protein/lactate-containing surface layer. Such a layer can be thicker in an effective electrochemical sense but less compact and less protective. Therefore, R_pl_ remains the more direct indicator of barrier performance, while d_eff_ should be interpreted together with Q, n, and the overall impedance response.

Among the three materials, TNZS consistently showed the highest R_pl_ in all media. In PBS, its R_pl_ was about 3.4 times higher than CP-Ti and 1.7 times higher than Ti–6Al–4V. In the inflammatory medium, TNZS showed R_pl_ values about 2.2 times higher than CP-Ti and 1.5 times higher than Ti–6Al–4V. In the severe inflammatory medium, TNZS still retained an R_pl_ about 2.8 times higher than CP-Ti and 1.6 times higher than Ti–6Al–4V. This confirms that the beneficial effect of the TNZS alloy composition was preserved even after one year of immersion.

#### 3.1.3 Potentiodynamic Polarization

Figure 4 shows the potentiodynamic polarization (PDP) curves of CP-Ti, Ti–6Al–4V, and TNZS after 372 d of immersion in normal, inflammatory, and severe inflammatory media. The corresponding electrochemical parameters extracted by Tafel extrapolation are summarized in Supplementary Table ST3. The polarization curves clearly show that both the alloy composition and the chemical nature of the immersion medium strongly affected the corrosion response after long-term exposure.

**Figure 4:**
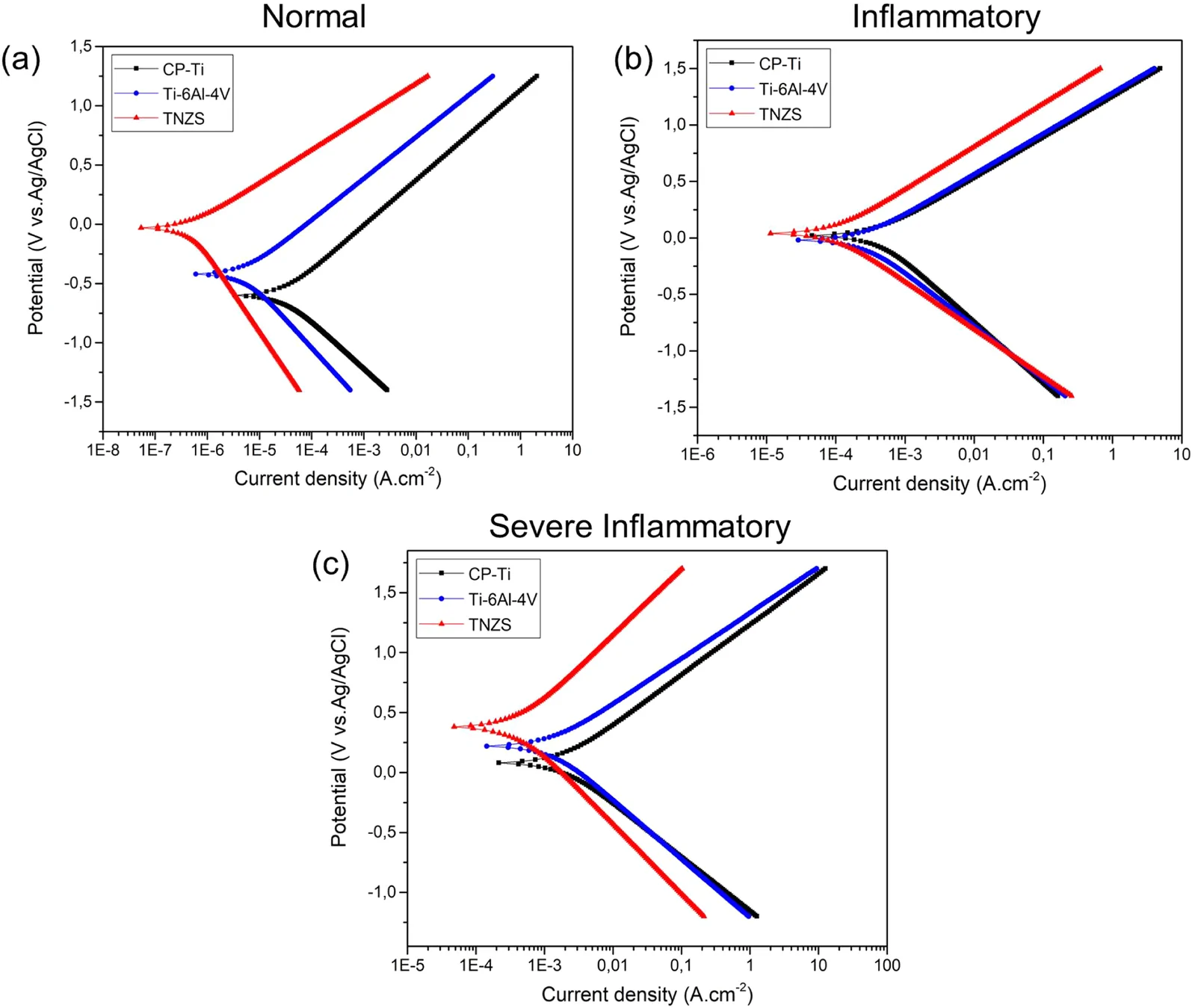
Potentiodynamic polarization curves of CP-Ti, Ti–6Al–4V, and TNZS after 372 d of immersion in different simulated physiological conditions: (a) normal PBS, (b) inflammatory PBS/H_2_O_2_/HCl solution, and (c) severe inflammatory PBS/H_2_O_2_/BSA/lactate/HCl solution.

After one year in normal condition, all materials still showed lower corrosion current densities than in the inflammatory media, confirming that neutral PBS remained the least aggressive condition. However, compared with the 12 h results reported previously, the one-year i_corr_ values increased for all specimens. The i_corr_ values were 3.90 ± 0.45 μA·cm^–2^ for CP-Ti, 0.54 ± 0.08 μA·cm^–2^ for Ti–6Al–4V, and 0.050 ± 0.008 μA·cm^–2^ for TNZS. This indicates that, although PBS promotes passive film formation during short-term immersion, long-term static exposure can still lead to passive-layer aging, hydration, defect formation, or accumulation of surface corrosion products. Therefore, the long-term PBS response should not be interpreted as continuous improvement of passivity, but rather as formation of a stabilized yet less ideal passive surface.

The corrosion tendency in normal PBS followed the order CP-Ti > Ti–6Al–4V > TNZS. TNZS maintained the lowest i_corr_, indicating that its passive layer retained superior barrier properties after prolonged immersion. The E_corr_ values were −0.60 V for CP-Ti, −0.42 V for Ti–6Al–4V, and −0.03 V for TNZS versus Ag/AgCl. The nobler E_corr_ of TNZS suggests a more stable electrochemical surface state, although the lower i_corr_ is the stronger indication of improved corrosion resistance.

In the inflammatory medium, the corrosion current density increased markedly for all materials. The i_corr_ values were much higher than those obtained for the specimens aged in PBS, showing that surfaces aged in the initially acidic H_2_O_2_-containing formulation were more susceptible to corrosion during re-exposure to the fresh corresponding test medium. Compared with the 12 h values of the previous study, the one-year inflammatory i_corr_ values were also slightly higher, indicating cumulative passive-film degradation. The long-term surface modification may have resulted from reactions occurring during the early stages of exposure, progressive solution aging, passive-film hydration and dissolution, adsorption or degradation of solution species, and accumulation of released products. Since residual H_2_O_2_ was not measured, its contribution over the entire 372 d period cannot be quantified.

The increase in i_corr_ in the inflammatory medium can be attributed to the combined effects of low pH and H_2_O_2_. Hydrogen peroxide can participate in cathodic reactions and generate reactive peroxide-related species on titanium surfaces, while acidic conditions facilitate oxide dissolution. For CP-Ti, this mainly reflects weakening of the TiO_2_-based passive film. For Ti–6Al–4V, instability or dissolution of Al- and V-containing oxides may further increase surface heterogeneity. TNZS also showed increased i_corr_ in this medium, but its corrosion current density remained lower than those of CP-Ti and Ti–6Al–4V, confirming its better resistance to peroxide-assisted corrosion.

The severe inflammatory medium produced the highest i_corr_ values among all conditions. After 372 d, i_corr_ values were substantially higher than those measured in PBS and inflammatory media, confirming that severe inflammatory conditions caused the strongest passive-layer degradation. This trend is consistent with the EIS results, where the passive-layer resistance decreased after one year. The severe inflammatory medium initially contained low pH, H_2_O_2_, BSA, and lactate. These constituents may interact synergistically, particularly during the initial exposure period; however, their concentrations and chemical states after prolonged static immersion were not determined. Low pH increases oxide solubility, H_2_O_2_ promotes oxidative attack, lactate may complex with dissolved titanium species, and BSA adsorption can modify the surface reactions and produce a heterogeneous interfacial layer.

The E_corr_ values in the inflammatory and severe inflammatory media shifted to more electropositive values compared with PBS. This reflects the oxidizing nature of the H_2_O_2_-containing solutions. However, E_corr_ alone cannot be used as a direct measure of corrosion resistance. A positive potential shift in oxidizing media can occur at the same time as an increase in corrosion current density. Therefore, the simultaneous evaluation of E_corr_ and i_corr_ is essential. TNZS showed the most positive E_corr_ in the severe inflammatory medium but also the lowest i_corr_, indicating that it remained in a nobler passive state while maintaining slower dissolution kinetics than CP-Ti and Ti–6Al–4V.

The cathodic Tafel slopes also changed with the environment, reflecting differences in cathodic reaction kinetics. In PBS, the higher |β_c_| of TNZS suggests slower cathodic kinetics on its more protective surface. In the inflammatory and severe inflammatory media, the cathodic process was influenced not only by oxygen reduction but also by H_2_O_2_ reduction, adsorbed intermediates, protein/lactate adsorption, and changes in passive-film chemistry. Minor fluctuations observed in the aggressive-media branches can be attributed to surface heterogeneity and repeated local dissolution/repassivation processes.

Overall, the PDP results confirm that long-term immersion reduced the corrosion resistance of all materials, as shown by increased i_corr_ after 372 d. The corrosion severity followed the order normal < inflammatory < severe inflammatory, while the material ranking remained CP-Ti > Ti–6Al–4V > TNZS in terms of corrosion current density. The superior performance of TNZS can be attributed to the presence of Nb, Zr, and Si, which promote the formation of a more stable passive film. From an implant perspective, the increased i_corr_ under inflammatory conditions indicates a greater tendency for ion release and surface degradation. This is particularly important for Ti–6Al–4V because corrosion may involve Al and V release. The lower i_corr_ of TNZS in all media therefore supports its potential advantage as a long-term dental implant material.

To provide an integrated comparison of the electrochemical response after short- and long-term exposure, the key EIS- and PDP-derived parameters are summarized as property maps in Figure 5. In Figure 5a, the relationship between corrosion current density, i_corr_, and passive-layer resistance, R_pl_, shows that increasing environmental aggressiveness shifts the materials toward higher i_corr_ and lower R_pl_, confirming progressive loss of passive-film protection from normal to inflammatory and severe inflammatory media. In Figure 5b, the E_corr_–β_c_ map shows that a more positive corrosion potential alone does not necessarily indicate improved corrosion resistance, particularly in the H_2_O_2_-containing media, where oxidizing conditions can shift E_corr_ positively while corrosion kinetics remain accelerated. In Figure 5c, the E_corr_–C_eff_ map further demonstrates changes in the electrochemically active passive layer, with higher C_eff_ generally reflecting a less protective or more defective interfacial state. Across all three maps, TNZS remains positioned toward lower i_corr_, higher R_pl_, and more stable passive-layer behavior compared with CP-Ti and Ti–6Al–4V, supporting the persistence of its superior corrosion resistance after one-year exposure.

**Figure 5:**
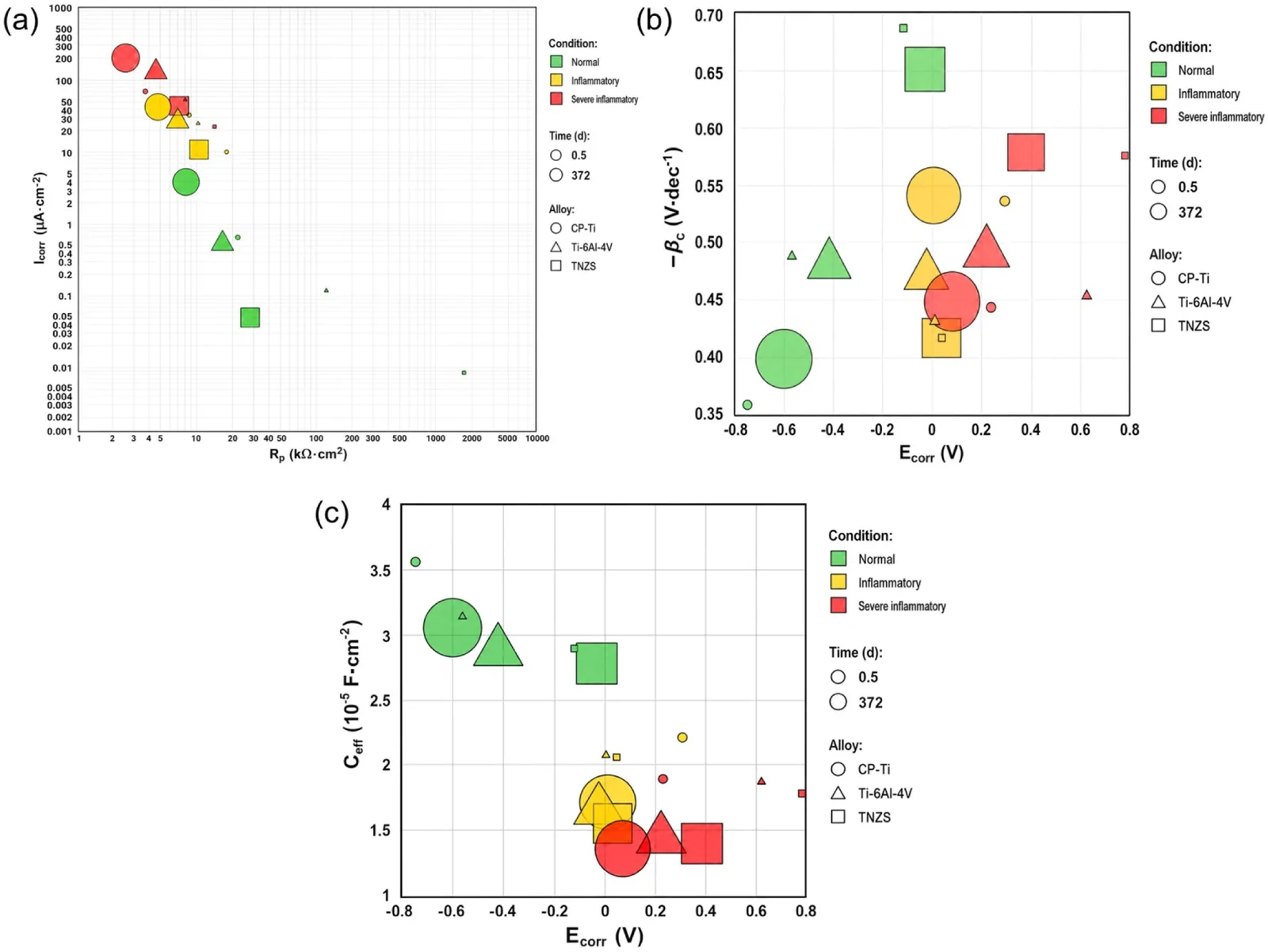
Integrated electrochemical-property maps of CP-Ti, Ti–6Al–4V, and TNZS after short-term exposure, 0.5 day (12 h), and long-term exposure, 372 d, in simulated normal, inflammatory, and severe inflammatory media: (a) corrosion current density, i_corr_, as a function of passive-layer resistance, R_pl_; (b) cathodic Tafel slope, −β_c_, as a function of corrosion potential, E_corr_; and (c) effective capacitance, C_eff_, as a function of E_corr_. Colors indicate the simulated condition, marker size indicates exposure time, and marker shape indicates alloy type. Lower i_corr_ combined with higher R_pl_ indicates a more protective passive state.

### 3.2 Elemental Release

The elemental release profiles of CP-Ti, Ti–6Al–4V, and TNZS after 372 d of immersion in the simulated normal, inflammatory, and severe inflammatory media are shown in Figure 6a–c. The corresponding numerical release values, and also the pH variations of each medium are listed in Supplementary Table ST4.

**Figure 6:**
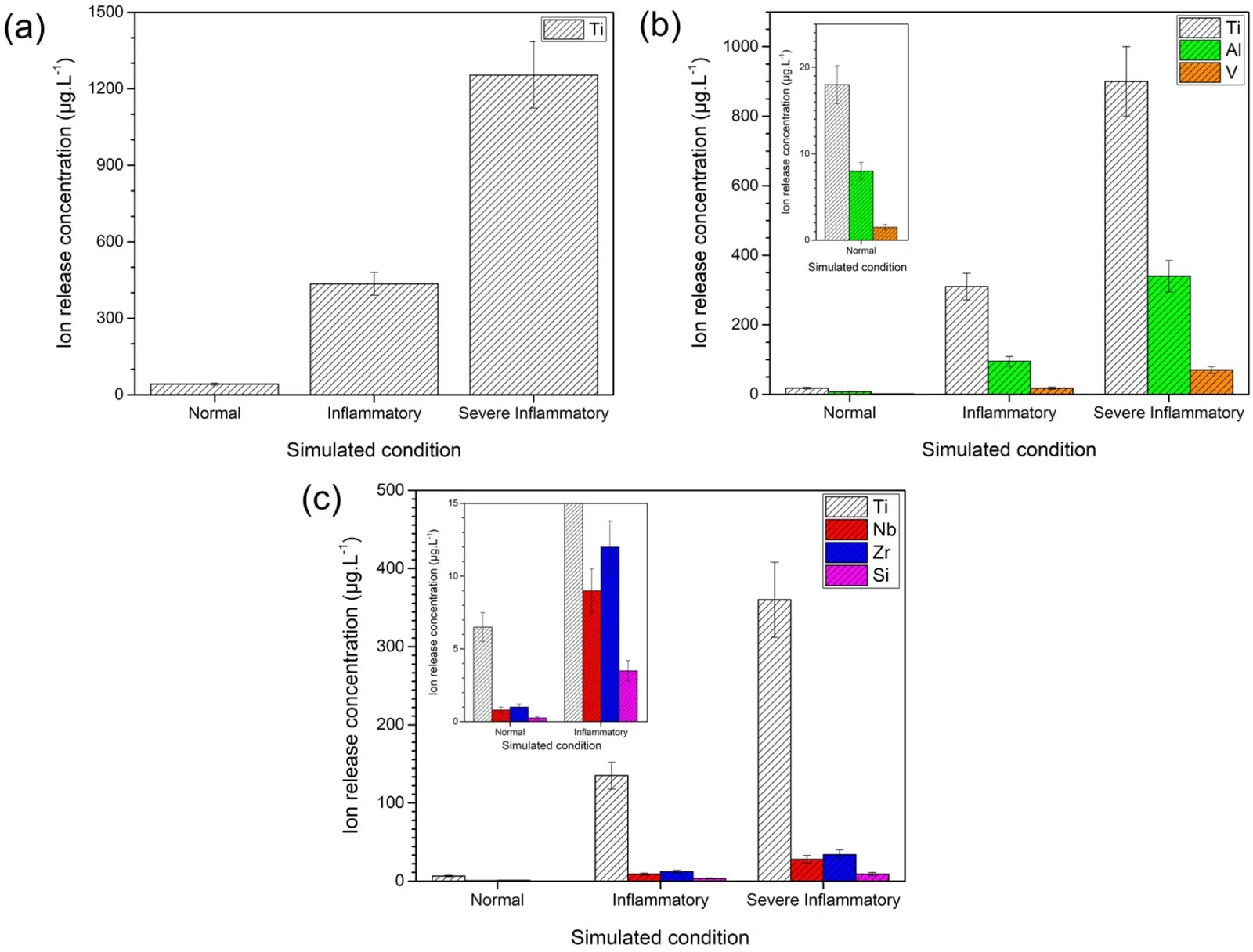
Elemental release from (a) CP-Ti, (b) Ti–6Al–4V, and (c) TNZS after 372 d of immersion in simulated normal, inflammatory, and severe inflammatory media. Insets in (b) and (c) show the low-concentration release data in normal and inflammatory media more clearly.

The visual appearance of the solutions after 372 d is presented in Supplementary Figure S3. While the normal solutions remained nearly colorless, the inflammatory and severe inflammatory media showed a pale yellow coloration, particularly for CP-Ti and Ti–6Al–4V. This color change is most likely related to the reaction between titanium-based passive films and H_2_O_2_, forming Ti–H_2_O_2_ or Ti­(IV)-peroxide complexes. Yellow gel or precipitate formation from Ti/H_2_O_2_ interactions has been reported previously by Tengvall et al.^36,37^ and Yu et al.,^11^ supporting the interpretation that the color change reflects peroxide-assisted titanium dissolution.

In normal PBS, all materials showed limited elemental release, confirming the protective role of the passive oxide film under near-neutral physiological conditions. CP-Ti released only Ti, while Ti–6Al–4V released Ti together with smaller amounts of Al and V. TNZS showed the lowest total release among the three materials. In this alloy, Ti was the main released element, whereas Nb, Zr, and Si were detected only at very low levels. This behavior agrees with previous long-term immersion studies, where Ti–6Al–4V in PBS/protein-containing media showed relatively limited ion release and a tendency toward stabilization after prolonged exposure due to oxide growth or precipitation of dissolved species on the surface. Karimi et al.^29^ reported that Ti release from Ti–6Al–4V increased at earlier immersion times but did not continue to increase after longer exposure, and Al was not detected by ICP-OES in their PBS/BSA system. Similarly, studies on Ti–Nb–Zr–Ta^30,31^ and TiZrNbFeMo^32^ alloys reported lower ion release than CP-Ti and Ti–6Al–4V, supporting the present low-release behavior of TNZS in PBS.

In the inflammatory medium, elemental release increased for all materials. This increase is consistent with the electrochemical results, where the H_2_O_2_-containing acidic medium reduced the corrosion resistance of the passive films. H_2_O_2_ can promote peroxide-assisted oxidation and partial dissolution of titanium oxide, while lower pH further destabilizes the passive layer. This trend is consistent with Höhn and Virtanen,^33^ who showed that Ti, Al, and V release from Ti–6Al–4V markedly increased under simulated inflammatory conditions at pH 5 in the presence of H_2_O_2_. In their work, Al and V release reached the mg·L^–1^ or sub-mg·L^–1^ range depending on surface condition and immersion time, which is comparable in order of magnitude to the present Ti–6Al–4V release in aggressive media. In contrast, TNZS released lower total amounts of elements than both conventional materials, and its alloying-element release followed the order Zr ≥ Nb > Si, indicating better passive-film stability.

The severe inflammatory medium produced the highest elemental release for all materials. This environment combines low pH, H_2_O_2_, BSA, and lactate, which together can accelerate passive-film degradation. Previous studies on albumin-containing solutions showed that proteins can alter oxide chemistry, interact with released ions, and modify dissolution/precipitation processes during long-term immersion.^34^ The present results support this behavior: CP-Ti showed a strong increase in Ti release, while Ti–6Al–4V released substantial amounts of Ti, Al, and V. TNZS also showed increased release compared with PBS and the inflammatory medium, but its total release remained much lower than CP-Ti and Ti–6Al–4V. This confirms that TNZS was more resistant to long-term degradation even in the most aggressive simulated biological condition.

The final pH measurements showed that PBS remained close to neutral, whereas the inflammatory and severe inflammatory media remained acidic after 372 d. However, pH alone does not define the full chemistry of the aged solutions. In particular, the residual H_2_O_2_ concentration, dissolved oxygen, ORP, protein degradation, lactate stability, and possible evaporation were not measured. Therefore, the final pH values confirm only that the media retained distinct acidity ranges at the end of exposure.

The elemental release results also support the better biological response previously observed for TNZS compared with Ti–6Al–4V.^15^ This behavior can be partly attributed to its lower total ion release and more favorable released species: TNZS does not release Al or V, while Nb and Zr are generally regarded as more biocompatible β-Ti alloying elements. The low Si release is also consistent with the small Si content and the role of silicide/intermetallic phases in improving corrosion resistance and retaining alloying elements within the Ti-based matrix. This interpretation is also supported by previous work on metallurgically alloyed Ti-based biomaterials, where stable intermetallic/matrix incorporation limited detectable alloying-element release by ICP-OES while maintaining cytocompatibility.^35^ Although the absolute released masses after one year are expected to be low compared with general systemic exposure levels, corrosion products are released locally at the tissue–implant interface, where even small amounts may become relevant under chronic inflammation, or mechanically assisted corrosion. Therefore, the lower release from TNZS, together with the absence of Al and V, suggests a reduced risk of local ion accumulation and inflammatory feedback, which is consistent with its improved cell response.

### 3.3 Limitations of the Study

A limitation of this study is that the long-term immersion media were not chemically monitored throughout the 372 d exposure. The solutions were static and non-refreshed, and only the final pH was measured. Residual H_2_O_2_ concentration, dissolved oxygen, oxidation–reduction potential, protein degradation, lactate stability, and possible evaporation were not quantified. Therefore, the study does not establish that the specimens were exposed to a constant inflammatory oxidizing environment for one year, and partial H_2_O_2_ depletion during the static exposure cannot be excluded. In addition, the experiments were performed at ambient temperature rather than at physiological temperature, which may have affected corrosion kinetics, H_2_O_2_ stability, protein behavior, and ion-release rates. The immersion test also did not include mechanical loading, fretting, wear, or tribocorrosion, although such mechanically assisted degradation processes are relevant for dental implants. Furthermore, ICP-OES was performed on filtered solutions; therefore, the reported elemental release values represent filtered dissolved species and may underestimate total material release if corrosion particles, detached oxide debris, precipitates, or protein-bound species were retained by the filter. Another limitation is the absence of post-immersion surface chemical characterization, such as XPS which prevents direct confirmation of the long-term passive-film composition and enrichment in Nb, Zr, or Si. Accordingly, the results should be interpreted as long-term static exposure to media initially formulated to simulate normal, inflammatory, and severe inflammatory conditions, followed by standardized electrochemical re-exposure of aged surfaces to freshly prepared media. This approach allows comparison among CP-Ti, Ti–6Al–4V, and TNZS under controlled post-exposure testing conditions, but it does not reproduce the exact in-situ electrochemical state of the aged metal/solution system at day 372.

## 4 Conclusions

This study evaluated the long-term electrochemical behavior and elemental release of CP-Ti, Ti–6Al–4V, and TNZS after 372 d of immersion in simulated normal, inflammatory, and severe inflammatory environments. The results showed that the solution chemistry strongly affected passive-layer stability and ion release. Specimens aged in media initially formulated to simulate inflammatory and severe inflammatory conditions showed poorer post-exposure electrochemical performance and greater filtered elemental release than those aged in normal PBS. These differences reflect the cumulative effects of the initial solution chemistry and its subsequent evolution during static, non-refreshed exposure; they cannot be attributed to continuously maintained concentrations of H_2_O_2_, BSA, or lactate. Among the tested materials, TNZS consistently exhibited the most favorable corrosion response, including nobler open-circuit potential, lower corrosion current density, and higher passive-layer resistance.

ICP-OES analysis also showed that TNZS released the lowest total amount of elements in all media. Importantly, the absence of Al and V in TNZS eliminates Al- and V-related elemental release from the alloy, whereas the release of these elements remains a concern for Ti–6Al–4V during long-term implantation. Although all released concentrations remained relatively low, local ion accumulation at the tissue–implant interface may be clinically relevant under chronic inflammatory conditions.

These findings indicate that TNZS retained better relative electrochemical stability than the conventional titanium materials under the specific aging and testing conditions employed. Nevertheless, the results are limited to a comparative coupon-level study involving static, non-refreshed media and an accelerated chemical challenge. TNZS may therefore be considered a promising Al- and V-free candidate for further implant-related investigation.

## Supplementary Material



## Data Availability

The data supporting the findings of this study are available within the article.

## Notes

TNZS microstructure and phase characterization (Figure S1); EIS equivalent circuit (Figure S2); photographs of immersion solutions after 372 d (Figure S3); simulated solution compositions and pH (Table ST1); fitted EIS parameters (Table ST2); PDP parameters (Table ST3); elemental release and pH data (Table ST4) (PDF)
