# Supplementary material for: Corrosion Behavior of Ti–Nb–Zr–Si Alloy after One-Year Exposure to Simulated Inflammatory Conditions
Source: ACS Omega. 2026 Sep 24;11(39):60084–95. doi: 10.1021/acsomega.6c07507 (PMC13647382; doi:10.1021/acsomega.6c07507)
Supplement: Supplementary file 1 [file ao6c07507_si_001.pdf]

Corrosion behavior of Ti–Nb–Zr–Si alloy after one-year exposure to simulated inflammatory conditions

Aydin Bordbar-Khiabani [1,*], Michael Gasik [2]

[1] VTT Technical Research Centre of Finland Ltd., Tekniikantie 21, 02044 Espoo, Finland

[2] Department of Chemical and Metallurgical Engineering, School of Chemical Engineering, Aalto University, 02150 Espoo, Finland

*Corresponding author's email: aidinbordbar@gmail.com, aydin.bordbar-khiabani@vtt.fi

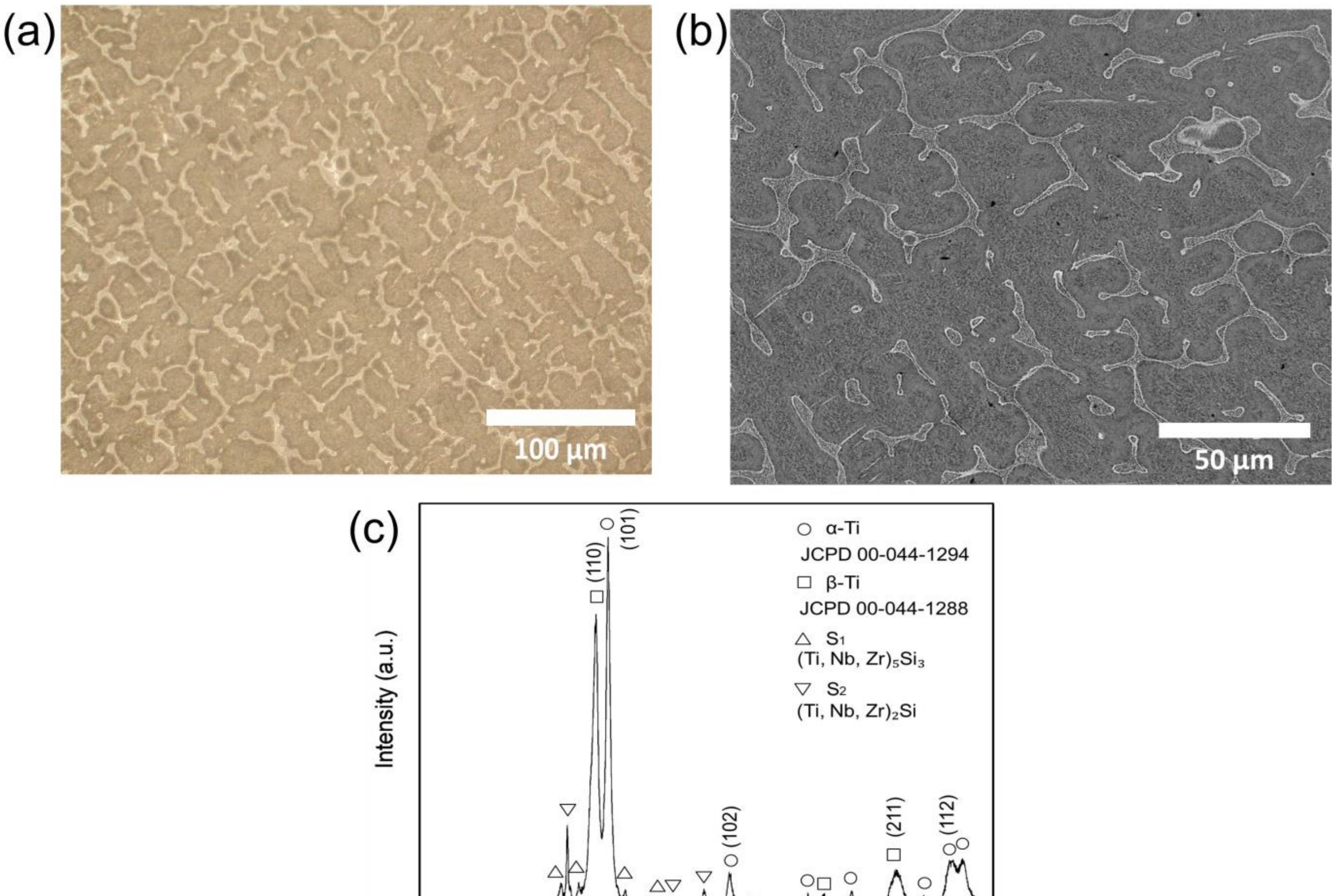

Figure S1. (a) Optical microscopy image, (b) back-scattered scanning electron microscopy micrograph, (c) X-ray diffraction pattern of the TNZS alloy [15].

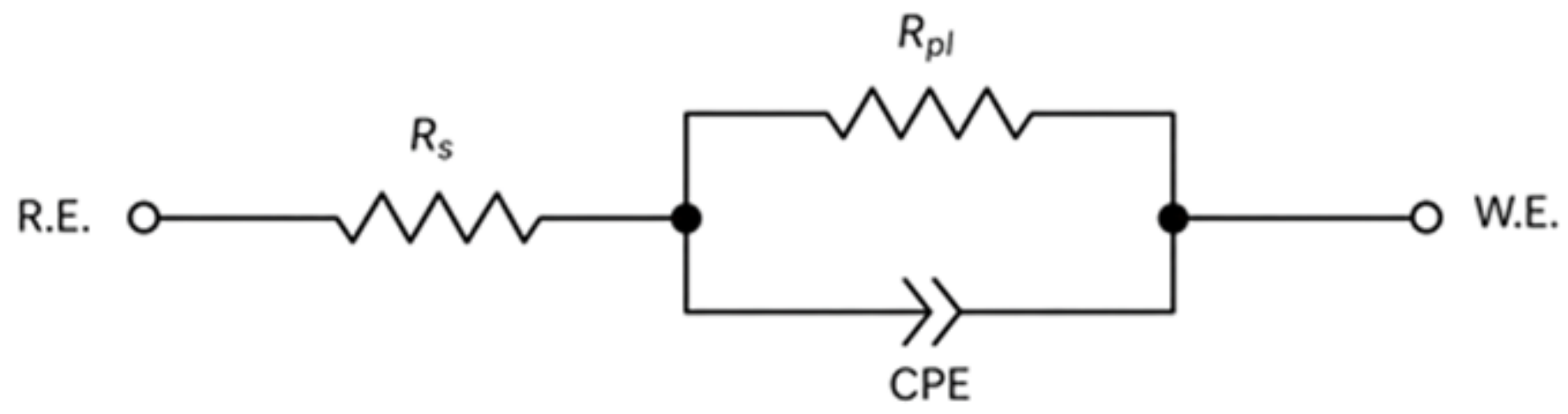

Figure S2. The equivalent circuit employed to fit the EIS data in Figures 2 and 3.

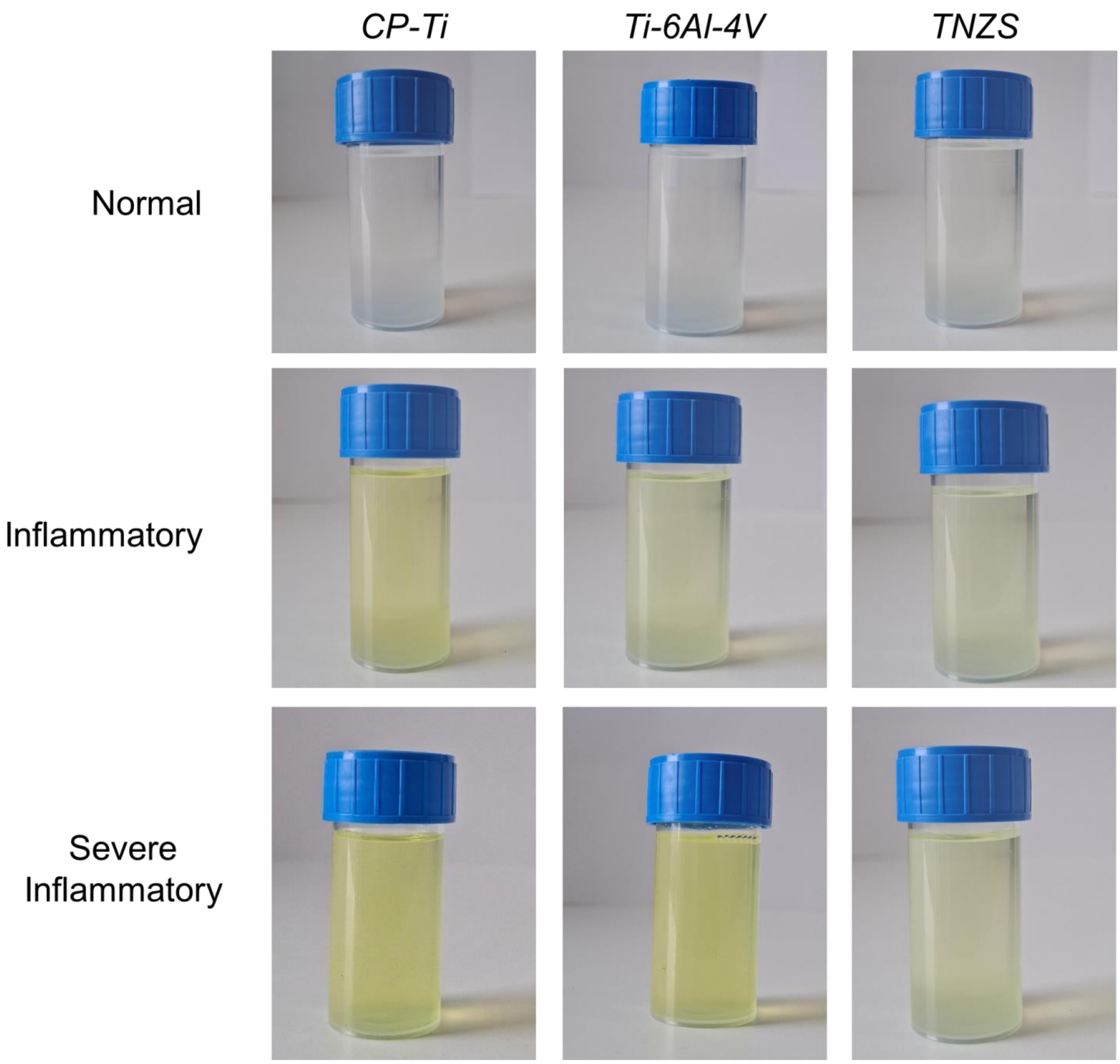

Figure S3. Representative photographs of the immersion solutions after 372 d of exposure to CP-Ti, Ti–6Al–4V, and TNZS specimens in normal, inflammatory, and severe inflammatory media.

Table ST1. Identification names for the simulated solutions with their respective composition, and pH.

Condition	Reagent and amount					pH
	PBS	H_2O_2	HCl	BSA*	CLH**	
Normal	5 standard tablets in 1 L deionized water	-	-	-	-	7.4±0.1
Inflammatory		150 mM	50 mM	-	-	5.2±0.1
Severe inflammatory				10 $g \cdot L^{-1}$	10 $g \cdot L^{-1}$	3.0±0.2

* Bovine serum albumin

** Calcium l-lactate hydrate

Table ST2. Parameters determined from fitting of the EIS plots of the CP-Ti, Ti-6Al-4V, and TNZS after 372 d exposure in different simulated conditions.

Condition	Specimen	R_{pl} ($k\Omega \cdot cm^2$)	CPE (10^{-5} $\Omega^{-1} \cdot cm^{-2} \cdot S^n$)	n	C_{eff} (10^{-5} $F \cdot cm^{-2}$)	d_{eff} (nm)
Normal	CP-Ti	8.200 ± 0.420	3.340 ± 0.226	0.940 ± 0.018	3.036 ± 0.193	1.312 ± 0.074
	Ti-6Al-4V	16.500 ± 0.760	3.050 ± 0.174	0.940 ± 0.023	2.868 ± 0.156	1.388 ± 0.096
	TNZS	28.260 ± 1.250	2.800 ± 0.168	0.950 ± 0.017	2.764 ± 0.162	1.441 ± 0.085
Inflammatory	CP-Ti	4.850 ± 0.310	2.250 ± 0.194	0.890 ± 0.027	1.709 ± 0.151	2.330 ± 0.171
	Ti-6Al-4V	7.020 ± 0.390	2.050 ± 0.147	0.900 ± 0.034	1.652 ± 0.118	2.410 ± 0.190
	TNZS	10.500 ± 0.536	1.820 ± 0.152	0.910 ± 0.026	1.545 ± 0.133	2.577 ± 0.214
Severe inflammatory	CP-Ti	2.670 ± 0.238	2.350 ± 0.224	0.840 ± 0.036	1.380 ± 0.143	2.886 ± 0.260
	Ti-6Al-4V	4.610 ± 0.327	2.100 ± 0.169	0.860 ± 0.043	1.435 ± 0.113	2.774 ± 0.241
	TNZS	7.200 ± 0.468	1.850 ± 0.158	0.880 ± 0.032	1.405 ± 0.104	2.834 ± 0.229

Table ST3. PDP parameters of the CP-Ti, Ti-6Al-4V, and TNZS after 372 d exposure in different simulated conditions.

Condition	Specimen	E_{corr} (V vs. Ag/AgCl)	$-\beta_c$ (V·dec^{-1})	i_{corr} (μA·cm^{-2})
Normal	CP-Ti	−0.5923 ± 0.0337	0.4078 ± 0.0368	3.90 ± 0.45
	Ti-6Al-4V	−0.4216 ± 0.0264	0.4865 ± 0.0417	0.54 ± 0.08
	TNZS	−0.0347 ± 0.0131	0.6428 ± 0.0526	0.050 ± 0.008
Inflammatory	CP-Ti	0.0248 ± 0.0193	0.5326 ± 0.0473	41.36 ± 4.72
	Ti-6Al-4V	−0.0167 ± 0.0152	0.4764 ± 0.0362	27.64 ± 3.18
	TNZS	0.0442 ± 0.0127	0.4278 ± 0.0331	10.72 ± 1.27
Severe inflammatory	CP-Ti	0.0861 ± 0.0214	0.4563 ± 0.0425	198.40 ± 21.36
	Ti-6Al-4V	0.2147 ± 0.0286	0.4971 ± 0.0468	132.60 ± 14.27
	TNZS	0.3728 ± 0.0368	0.5736 ± 0.0487	45.18 ± 5.16

Table ST4. Elemental release from CP-Ti, Ti-6Al-4V, and TNZS and pH variations after 372 d exposure in different simulated conditions. Release values are reported in μg·L^{-1}.

Condition	Specimen	Ti	Al	V	Nb	Zr	Si	pH
Normal	CP-Ti	42.15 ± 4.83	-	-	-	-	-	7.38 ± 0.53
	Ti-6Al-4V	18.29 ± 2.24	8.06 ± 0.92	1.50 ± 0.37	-	-	-	7.36 ± 0.24
	TNZS	6.09 ± 0.70	-	-	0.80 ± 0.18	1.07 ± 0.23	0.25 ± 0.08	7.34 ± 0.33
Inflammatory	CP-Ti	420.75 ± 43.70	-	-	-	-	-	5.25 ± 0.16
	Ti-6Al-4V	310.48 ± 38.40	95.40 ± 14.20	18.20 ± 3.25	-	-	-	5.22 ± 0.25
	TNZS	135.30 ± 17.30	-	-	9.20 ± 1.53	12.30 ± 1.72	3.50 ± 0.76	5.17 ± 0.18
Severe inflammatory	CP-Ti	1250.00 ± 130.80	-	-	-	-	-	3.32 ± 0.11
	Ti-6Al-4V	912.00 ± 96.40	340.55 ± 45.70	70.30 ± 9.60	-	-	-	3.29 ± 0.09
	TNZS	360.27 ± 46.30	-	-	28.40 ± 5.16	34.20 ± 5.80	9.50 ± 2.86	3.22 ± 0.17
